# Intermittent bolus feeding does not enhance protein synthesis, myonuclear accretion, or lean growth more than continuous feeding in a premature piglet model

**DOI:** 10.1152/ajpendo.00236.2021

**Published:** 2021-11-01

**Authors:** Marko Rudar, Jane K. Naberhuis, Agus Suryawan, Hanh V. Nguyen, Barbara Stoll, Candace C. Style, Mariatu A. Verla, Oluyinka O. Olutoye, Douglas G. Burrin, Marta L. Fiorotto, Teresa A. Davis

**Affiliations:** ^1^United States Department of Agriculture/Agricultural Research Service Children’s Nutrition Research Center, Department of Pediatrics, Baylor College of Medicine, Houston, Texas; ^2^Department of Animal Sciences, Auburn University, Auburn, Alabama; ^3^The Department of Pediatric Surgery, Nationwide Children’s Hospital, Columbus, Ohio; ^4^Michael E. DeBakey Department of Surgery, Baylor College of Medicine, Houston, Texas

**Keywords:** feeding modality, myonuclear accretion, prematurity, protein synthesis, skeletal muscle

## Abstract

Optimizing enteral nutrition for premature infants may help mitigate extrauterine growth restriction and adverse chronic health outcomes. Previously, we showed in neonatal pigs born at term that lean growth is enhanced by intermittent bolus compared with continuous feeding. The objective was to determine if prematurity impacts how body composition, muscle protein synthesis, and myonuclear accretion respond to feeding modality. Following preterm delivery, pigs were fed equivalent amounts of formula delivered either as intermittent boluses (INT; *n* = 30) or continuously (CONT; *n* = 14) for 21 days. Body composition was measured by dual-energy X-ray absorptiometry (DXA) and muscle growth was assessed by morphometry, myonuclear accretion, and satellite cell abundance. Tissue anabolic signaling and fractional protein synthesis rates were determined in INT pigs in postabsorptive (INT-PA) and postprandial (INT-PP) states and in CONT pigs. Body weight gain and composition did not differ between INT and CONT pigs. Longissimus dorsi (LD) protein synthesis was 34% greater in INT-PP than INT-PA pigs (*P* < 0.05) but was not different between INT-PP and CONT pigs. Phosphorylation of 4EBP1 and S6K1 and eIF4E·eIF4G abundance in LD paralleled changes in LD protein synthesis. Satellite cell abundance, myonuclear accretion, and fiber cross-sectional area in LD did not differ between groups. These results suggest that, unlike pigs born at term, intermittent bolus feeding does not enhance lean growth more than continuous feeding in pigs born preterm. Premature birth attenuates the capacity of skeletal muscle to respond to cyclical surges in insulin and amino acids with intermittent feeding in early postnatal life.

**NEW & NOTEWORTHY** Extrauterine growth restriction often occurs in premature infants but may be mitigated by optimizing enteral feeding strategies. We show that intermittent bolus feeding does not increase skeletal muscle protein synthesis, myonuclear accretion, or lean growth more than continuous feeding in preterm pigs. This attenuated anabolic response of muscle to intermittent bolus feeding, compared with previous observations in pigs born at term, may contribute to deficits in lean mass that many premature infants exhibit into adulthood.

## Graphical abstract

**Figure:**
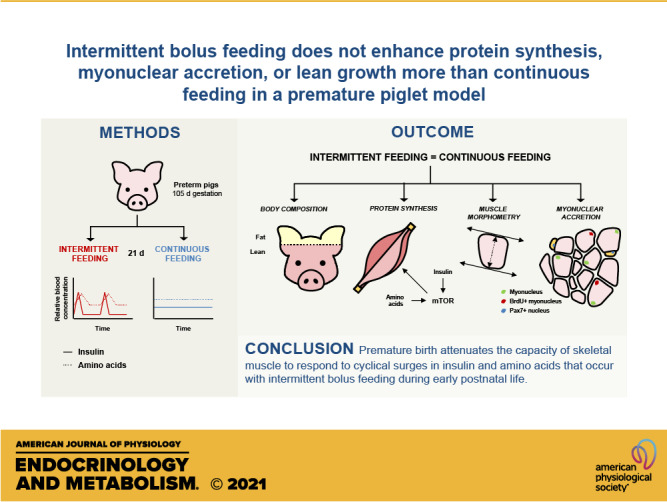

## INTRODUCTION

The incidence of preterm birth in the United States is increasing and accounts for ∼1 in 10 births (1). Despite advancements in perinatal medicine, premature infants are still at high risk for poor growth outcomes and are frequently discharged weighing less than the 10th percentile for age (2). Postnatal growth failure is associated with adverse long-term health outcomes including impaired cognitive and neurological development, compromised motor function, less skeletal muscle mass, and increased risk for developing obesity, insulin resistance, and heart disease (3–5). Current recommendations for premature infants target an extrauterine growth rate that approximates the estimated intrauterine growth rate of a healthy fetus at the same postconception age (6). However, the immature gastrointestinal function of premature infants limits the rapid advancement of enteral feeding and may lead to persistent deficits in energy and protein intake that preclude adequate growth (6–9). At term equivalent age, premature infants tend to have lower body weight, less lean mass, and increased adiposity than infants born at term (10). Strategies to promote lean growth while minimizing fat accretion in premature infants are needed.

An important objective for premature infants receiving parenteral nutrition is the introduction and advancement of enteral feeding because it minimizes complications linked to prolonged parenteral feeding and supports optimal growth. In infants unable to coordinate breathing, suckling, and swallowing, nasogastric or orogastric tube feeding is often needed to provide enteral nutrition. An important consideration for tube feeding premature infants is whether to feed them continuously or on an intermittent bolus schedule. The primacy of either continuous or intermittent bolus feeding modalities has been debated, particularly in the context of body weight gain and morbidity outcomes. Although a recent Cochrane systematic review concluded that there is insufficient evidence regarding the clinical benefits of intermittent bolus versus continuous feeding to recommend one feeding strategy over another (11), the impact of feeding modality on lean growth and body composition in a premature infant population has not yet been evaluated.

Insulin and amino acids independently activate the mechanistic target of rapamycin (mTOR) complex 1 (mTORC1) signaling pathway that integrates nutrient, hormone, and stress signals to regulate translation initiation and protein synthesis (12–14). Skeletal muscle, the fastest growing protein pool in neonates, is exceptionally sensitive to these anabolic signals derived from insulin and amino acids (15). The incorporation of new myonuclei into muscle fibers, derived from the proliferation and differentiation of satellite cells (SC), is also a key determinant of muscle growth (16). In the neonatal pig, a highly translatable animal model for infants (17, 18), intermittent bolus feeding elicits cyclical surges in insulin and amino acids. These surges simulate a physiological feeding and fasting pattern normally seen in term infants that potentiates skeletal muscle protein synthesis (19–21). Continuous feeding, on the other hand, is associated with low and constant levels of circulating hormones and nutrients. Although continuous feeding may improve feeding tolerance and glycemic stability, the absence of recurring surges in insulin and amino acids may limit muscle protein synthesis and consequently lean growth in the neonate (19–22). Indeed, prolonged intermittent bolus feeding promotes body weight gain and increases lean mass compared with continuous feeding in neonatal pigs born at term (23, 24).

Recently, our research group demonstrated that the activation of insulin and amino acid signaling upstream of mTORC1, translation initiation factor signaling downstream of mTORC1, and protein synthesis in skeletal muscle in response to feeding are diminished in preterm compared with full-term neonatal pigs, and this blunted response contributes to reduced weight gain (25). Moreover, our recent hyperinsulinemic-euaminoacidemic-euglycemic and euinsulinemic-hyperaminoacidemic-euglycemic clamp studies demonstrated that mTORC1 signaling and protein synthesis in skeletal muscle of preterm pigs are blunted in response to equivalent increases in both insulin and amino acids (26). Considering this blunted anabolic response to feeding, it is unclear whether pigs born preterm can respond to intermittent bolus and continuous feeding modalities in the same manner as pigs born at term. Furthermore, the contribution of SCs to muscle growth following preterm birth, and potential interactions with feeding modality, is largely unexplored. The objectives of this study were to determine how intermittent bolus and continuous feeding modalities affect skeletal muscle protein synthesis, SC abundance, and myonuclear accretion in a preterm neonatal pig model and to delineate the contribution of muscle protein synthesis and SC activity to alterations in growth and body composition. We hypothesized that the lower lean growth rates of preterm neonates are offset by administering feeds with an intermittent bolus rather than a continuous modality.

## MATERIALS AND METHODS

### Animals and Surgery

The experimental protocol (AN-636) was approved by the Institutional Animal Care and Use Committee, Baylor College of Medicine. The protocol was conducted in compliance with the National Research Council’s *Guide for the Care and Use of Laboratory Animals*, and all surgical procedures described were performed under general anesthesia using sterile technique. Sows were obtained from a commercial farm, housed at the United States Department of Agriculture/Agricultural Research Service (USDA/ARS) Comparative Nutrition Research Facility (Houston, TX), and provided ad libitum access to feed (Laboratory Porcine Grower Diet 5084, LabDiet, St. Louis, MO; metabolizable energy, 3,160 kcal/kg diet; crude protein, 160 g/kg diet) and water for 1 wk before study procedures.

At *gestation day 105* (term is 114 days), piglets (Yorkshire × Landrace × Duroc × Hampshire; initial body weight, 952 ± 205 g; range, 600–1,488 g) from five pregnant sows were delivered by Caesarian section, as described previously (25, 27). This gestational age in pigs corresponds to a gestational age between 30 and 32 wk in humans (27). Following resuscitation, the piglets were temporarily housed in groups in heated plexiglass incubators before placement of an umbilical artery catheter (3.5 Fr, polyurethane, 15 in., single lumen) and an orogastric tube (5 Fr, polyvinyl chloride, 36 in.). Piglets were housed individually between 29°C and 32°C and maintained on a 12-h light/12-h dark cycle. All piglets received iron dextran (30 mg via intramuscular injection) and sterile sow plasma (4, 5, and 7 mL/kg at 6, 12, and 24 h, respectively, after birth via the umbilical artery catheter) to provide passive immunity. After birth, piglets were provided total parenteral nutrition via the umbilical artery catheter to meet or exceed the nutrient requirements of neonatal pigs for energy, amino acids, vitamins, and minerals [Table 1; formulation adapted from Stoll et al. (28)]. Piglets were monitored hourly for 5 days after birth with complete clinical evaluations (mentation, urination, defecation, vomiting, temperature, gait, respiration, skin color, mucus membrane perfusion, vocalization, and pain score) performed every 12 h. Institutional veterinarians were consulted when the assessed parameters were abnormal for neonatal pigs. After 7 days, the umbilical artery catheter and orogastric tube were removed and replaced with a jugular vein catheter, as described previously under general anesthesia and sterile technique (29), and a larger orogastric tube (8 Fr, polyvinyl chloride, 36 in.). Clinical evaluations of pigs were performed after the surgery as aforementioned. Jugular vein catheter patency was maintained with a continuous infusion of heparinized saline (1 mL·h^−1^ containing 0.5 U heparin per mL). All piglets were included in the studies unless they were not able to be resuscitated, did not maintain spontaneous breathing, did not survive the full duration of the experiment, or were euthanized on recommendation by an institutional veterinarian.

**Table 1.** Macronutrient composition of total parenteral nutrition.a,^b^

Ingredient	Content, g·L^−1^
Carbohydrate^c^	91.7
Lipid^d^	20.8
Amino acids^e^	66.7

^a^Electrolyte composition (g·L^−1^): calcium, 0.54; chloride, 1.28; potassium, 1.02; phosphate, 0.47; magnesium, 0.10; sodium, 0.67.

^b^Vitamin and trace minerals (mg·L^−1^): vitamin A, 1.375; vitamin D, 0.0104; vitamin E, 0.0123; vitamin K, 0.21; thiamine, 20.83; riboflavin, 1.04; niacin, 20.83; pantothenic acid, 4.17; pyridoxine, 2.09; biotin, 0.042; folic acid, 0.42; cyanocobalamin, 0.021; ascorbic acid, 208.3; iron, 26.25; zinc, 26.25; copper, 1.67; manganese, 1.04; selenium, 0.083; chromium, 0.042; iodine, 0.042.

^c^Carbohydrate supplied as dextrose.

^d^Lipid supplied as intralipid 20%; contains 20% soybean oil, 1.2% egg yolk phospholipids, 2.25% glycerin, and water for injection.

^e^Amino acids (g·L^−1^): alanine, 3.34; arginine, 2.84; aspartic acid, 5.14; cysteine·HCl, 1.49; glutamic acid, 6.43; glutamine, 5.14; glycine, 2.51; histidine, 1.67; isoleucine, 3.73; leucine, 6.62; lysine·HCl, 6.24; methionine, 1.67; phenylalanine, 3.41; proline, 4.82; serine, 3.60; threonine, 4.05; tryptophan, 0.77; tyrosine, 0.77; valine, 4.05.

### Experimental Design

Power analysis indicated that 15 pigs per treatment group were required to detect a 25% difference, with between-animal variation of 25%, a type I error of 0.05, and a power of 0.80. Pigs within sex were assigned at birth to either intermittent bolus (INT, *n* = 30; 7 male and 23 female) or continuous (CONT; *n* = 14, 5 male and 9 female) feeding modality treatments with the RANDBETWEEN function in Microsoft Excel 2016 (Microsoft Corporation, Redmond, WA). Pigs were assigned to the INT group with a 2-to-1 bias because the INT group was subdivided into a postabsorptive and postprandial group on the last day of the study (see later in this section for further detail). The milk replacer formula fed to the INT and CONT groups provided 210 kcal metabolizable energy·kg^−1^·day^−1^ and 16 g protein^−1^·kg^−1^·day^−1^ (Table 2). Vitamin and mineral content of the formula met or exceeded requirements for neonatal pigs (30).

**Table 2.** Ingredient and macronutrient composition of milk replacer formula

Ingredient, g·kg^−1^	
Whey protein concentrate^a^	109
Lactose	19.6
Corn oil	39.6
MCT oil^b^	25.8
Fat Pak 80^c^	13.8
Xanthan gum	0.6
Vitamin premix^d^	2.5
Mineral premix^d^	11.3
Water	778
Calculated nutrient content	
Total protein, g·kg^−1^	87.7
Total carbohydrate, g·kg^−1^	27.8
Total fat, g·kg^−1^	83.4
Energy, kcal·kg^−1^	1,173

^a^Whey protein concentrate supplied from NutraBio, Middlesex, NJ.

^b^Medium chain triglyceride oil.

^c^Fat Pak 80 supplied from Milk Specialties Global, Eden Prairie, MN.

^d^Vitamin and mineral premixes supplied from Dyets, Bethlehem, PA.

Piglets were advanced gradually from parenteral (at birth, *day 1*) to enteral feeding (*day 6*) over a 6-day period to mimic clinical treatment of premature infants that do not normally receive their full energy and protein requirements immediately after birth and to prevent the development of feeding intolerance and necrotizing enterocolitis (Table 3). Piglets in the INT group were fed every 3 h for the first 7 days (advancing from 6.75 to 22.5 mL·kg^−1^) and every 4 h thereafter (30 mL·kg^−1^); piglets in the CONT group were fed at a constant rate (advancing from 2.25 to 7.5 mL·kg^−1^·h^−1^) for the first 7 days and 7.5 mL·kg^−1^·h^−1^ thereafter. The meal duration for INT piglets decreased from 30 min on *day 2* to 15 min on *day 5*, which was maintained until the end of the experiment on *day 22* (equivalent to 12 days term-corrected age). Piglets were weighed every 2 days throughout the experiment, and parenteral and enteral nutrient intakes were adjusted according to the change in body weight.

**Table 3.** Calculated volume and nutrient intake from parenteral and enteral nutrition during the first 6 days after birth in pigs

	Volume,mL·kg^−1^·day^−1^	Protein,g·kg^−1^·day^−1^	Carbohydrate,g·kg^−1^·day^−1^	Fat,g·kg^−1^·day^−1^	Energy,kcal·kg^−1^·day^−1^
*Day 1*					
P^a^	144	9.6	13.2	3.0	114
E^b^	0	0	0	0	0
Sum^c^	144	9.6	13.2	3.0	114
*Day 2*					
P	120	8.0	11.0	2.5	95
E	54	4.8	1.5	4.5	63
Sum	174	12.8	12.5	7.0	158
*Day 3*					
P	144	9.6	13.2	3.0	114
E	72	6.4	2.0	6.0	84
Sum	216	16.0	15.2	9.0	198
*Day 4*					
P	96	6.4	8.8	2.0	76
E	108	9.6	3.0	9.0	126
Sum	204	16.0	11.8	11.0	202
*Day 5*					
P	60	4.0	5.5	1.3	48
E	135	12.0	3.8	11.3	158
Sum	195	16.0	9.3	12.6	206
*Day 6*+^d^					
P	0	0	0	0	0
E	180	16.0	5.0	15.0	210
Sum	180	16.0	5.0	15.0	210

^a^P, parenteral volume and macronutrient intake.

^b^E, enteral volume and macronutrient intake.

^c^Sum of parenteral and enteral volume and macronutrient intake.

^d^Volume and macronutrient intakes on *day 6* were maintained until the end of the study on *day 22*.

On *day 21*, all piglets were sedated with Telazol (2.2 mg/kg im) and Xylazine (1.1 mg/kg im) to determine body composition by dual-energy X-ray absorptiometry (DXA; Hologic QDR4500A, Marlborough, MA). Lean mass and fat mass data were adjusted according to previously published calibration equations for piglets (31). Two pigs from the INT group were excluded from analysis due to inadequate sedation for the DXA scan.

On *day 22* of the experiment, piglets in the INT group were subdivided randomly into postabsorptive (INT-PA, *n* = 14; 2 male and 12 female) and postprandial groups (INT-PP, *n* = 16; 5 male and 11 female). At the start of a 5-h period, the INT-PA group was fed at 0 h, the INT-PP group was fed at 0 h and 4 h, and the CONT group was fed at a constant rate for 5 h. Blood samples were collected into heparinized tubes immediately before feeding and at 30, 60, 90, 120, 180, and 240 min after feeding to determine a time-course of plasma glucose, insulin, and amino acid concentrations. Plasma was obtained after centrifugation at 10,000 *g* for 1 min and stored at −20°C until analyzed. At 4.5 h, all piglets were administered a flooding dose of L-Phe (1.50 mmol·kg^−1^ iv) containing L-[ring-^2^H_5_]Phe at 40 mol % (0.60 mmol·kg^−1^, Cambridge Isotope Laboratories, Inc., Tewksbury, MA). Tissue samples were collected from the longissimus dorsi (LD), gastrocnemius, soleus, heart, lung, brain, liver, jejunum, and pancreas immediately after euthanasia (Beuthanasia-D, 0.45 mL·kg^−1^ iv; Merck Animal Health, Kenilworth, NJ), snap-frozen in liquid nitrogen, and stored at −80°C until analyzed.

### Plasma Insulin, Glucose, and Branched-Chain Amino Acids

Plasma insulin concentrations were determined with a commercial kit (Porcine Insulin Radioimmunoassay no. PI-12K, MilliporeSigma, Burlington, MA). Plasma glucose concentrations were measured with the glucose oxidase method (Yellow Springs Instruments, model 2300, Yellow Springs, OH). Plasma branched-chain amino acid (BCAA) concentrations were analyzed by HPLC (PICO-TAG reverse phase column, Waters, Milford, MA) after protein removal and derivatization with phenylisothiocyanate, as described previously (32). Two pigs from the CONT group and three pigs from the INT group were excluded from analysis because their catheters lost patency and blood was not sampled.

### Tissue Fractional Protein Synthesis Rates

To determine tissue fractional protein synthesis rates (FSR), frozen tissue samples were homogenized on ice in 3 mL of 0.2 mol/L perchloric acid and centrifuged (3,000 *g* for 20 min). The supernatant containing the tissue-free amino acids was applied to a cation exchange resin (Dowex 50WX8, hydrogen form, 100–200 mesh, MilliporeSigma), washed with distilled water (3 × 3 mL), and eluted with 3 mL of 5 mol/L ammonium hydroxide. The pellet containing the tissue protein-bound amino acids was further washed with 0.2 mol/L perchloric acid (2 × 10 mL, 2 × 5 mL; each wash preceded with centrifugation at 3,000 *g* for 20 min) and hydrolyzed in 3 mL of 6 mol/L HCl at 110°C. The hydrolysate was then applied to the cation exchange resin and processed as aforementioned. Tissue-free and tissue protein-bound eluates were subsequently dried under vacuum, reconstituted in 0.1 mol/L sodium tetraborate, and derivatized with 20 mmol/L 5-(dimethylamino)-1-naphthalenesulfonyl chloride (in acetonitrile). After derivatization, samples were dried under vacuum and reconstituted in 0.1% formic acid for injection. Sample isotopic enrichment was analyzed on a triple quadrupole mass spectrometer (Thermo TSQ Vantage, Thermo Fisher Scientific, Waltham, MA) operating in positive electrospray ionization mode following chromatographic separation of amino acids (Accela 1200; Thermo Fisher Scientific). The (dimethylamino)-1-naphthalene sulphonamide derivatives of Phe were monitored at *m/z* 399 and 404 (daughter ion *m/z* 120 and 125) for L-Phe and L-[ring-^2^H_5_]Phe, respectively. Peak areas were converted to molar percentage enrichment (mol %) with calibration curves prepared from purified L-Phe and L-[ring-^2^H_5_]Phe. Tissue FSR was calculated as

$$FSR=\frac{E_{bound}\times 1,440\times 100\%}{E_{free}\times t}$$

where *FSR* (%/day) is defined as the proportion of protein synthesized per day, *E_bound_* is the enrichment of L-[ring-^2^H_5_]Phe in tissue protein-bound Phe (mol %) at time *t*, *E_free_* is the enrichment of L-[ring-^2^H_5_]Phe in tissue-free Phe (mol %), *t* (min) is the incorporation time of the tracer into tissue protein, and 1,440 is the min-to-day conversion factor. The isotopic enrichment of tissue-free Phe over the labeling period was corrected from the measured tissue value and the rate of decrease in plasma L-[ring-^2^H_5_]Phe isotopic enrichment from plasma samples collected 5, 15, and 30 min after injection of the tracer (33). Two pigs from the CONT group and three pigs from the INT group were excluded from this analysis because their catheters lost patency and the isotopic tracer was not administered.

### Eukaryotic Initiation Factor 4E·eIF4G Complex Abundance

Skeletal muscle was homogenized in homogenization buffer (20 mmol/L HEPES, 2 mmol/L EGTA, 50 mmol/L sodium fluoride, 100 mmol/L potassium chloride, and 0.2 mmol/L EDTA; pH 7.4) containing protease inhibitor cocktail (P3840; Millipore Sigma). Following centrifugation at 10,000 *g* at 4°C, samples were incubated with anti-eIF4E (1:10) overnight at 4°C. See Table 4 for comprehensive antibody information for immunoprecipitation and immunoblot analyses. The immunoprecipitate was recovered with BioMag goat anti-mouse IgG (Cat. No. 310007; Qiagen, Germantown, MD) blocked in 0.1% wt/vol nonfat dry milk in low-salt buffer (20 mmol/L Tris, 150 mmol/L sodium chloride, 5 mmol/L EDTA, 1% vol/vol Triton X-100, and 0.1% vol/vol β-mercaptoethanol; pH 7.4), washed twice with low-salt buffer, and washed once with a high-salt buffer (50 mmol/L Tris, 500 mmol/L sodium chloride, 5 mmol/L EDTA, 1% vol/vol Triton X-100, 0.5% wt/vol sodium deoxycholate, 0.1% wt/vol SDS, and 0.04% vol/vol β-mercaptoethanol; pH 7.4). After recovery of the immunoprecipitate, the sample was resuspended in 100 µL 1× Laemmli buffer, boiled for 5 min, and separated by SDS-PAGE on triple-wide gels; 10 µL immunoprecipitated samples (for eIF4E analysis) and 20 µL (for eIF4G analysis) were electrophoresed on separate gels. After wet transfer (10 mmol/L CAPS, pH 10.5; 10% methanol vol/vol) onto polyvinylidene difluoride membranes (PVDF; Amersham Hybond P Western Blotting Membrane, 0.45 µM; Cytiva, Marlborough, MA), blots were blocked in 5% wt/vol nonfat dry milk in tris-buffered saline with Tween20 (TBS-T; 0.1% vol/vol) for 1 h, incubated with anti-eIF4E (1:1,000) or anti-eIF4G (1:1,000) diluted in BSA (3% wt/vol in TBS-T) overnight at 4°C, incubated with the corresponding secondary antibody (1:5,000) diluted in nonfat dry milk (5% wt/vol in TBS-T) for 1 h at room temperature, and visualized with chemiluminescence (ECL Prime Western Blotting Detection Reagent; Cytiva) on ChemiDoc-It Imaging System (UVP, Upland, CA). Total eIF4G abundance was normalized to total eIF4E abundance.

**Table 4.** Antibody information for antibodies used in immunoprecipitation and immunoblot experiments

Antibody	Source	Identifier
*Primary*		
eIF4E (for immunoprecipitation)	Gift of Dr. Leanord Jefferson, Pennsylvania State University, College of Medicine, Hershey, PA	N/A
eIF4G	Millipore Sigma	Cat. No. 07-1800; RRID:AB_355697
eIF4E	Cell Signaling Technology	Cat. No. 9742; RRID:AB_823488
Raptor (for immunoprecipitation)	Cell Signaling Technology	Cat. No. 2280; RRID:AB_561245
Rheb	R&D Systems	Cat. No. MAB3246; RRID:AB_2178785
RagA	Cell Signaling Technology	Cat. No. 4357; RRID:AB_10545136
RagC	Cell Signaling Technology	Cat. No. 5466; RRID:AB_10692651
mTOR	Cell Signaling Technology	Cat. No. 2972; RRID:AB_330978
Mios (for immunoprecipitation)	Cell Signaling Technology	Cat. No. 13557; RRID:AB_2798254
Sestrin2	Cell Signaling Technology	Cat. No. 8487; RRID:AB_11178663
Mios	Proteintech Group	Cat. No. 20826-1-AP; RRID:AB_2878747
p-4EBP1 Thr70	Cell Signaling Technology	Cat. No. 9455; RRID:AB_330949
4EBP1	Bethyl Laboratories	Cat. No. A300-501A; RRID:AB_2277825
p-S6K1 Thr389	R&D Systems	Cat. No. AF8963; RRID:AB_355697
S6K1	Proteintech Group	Cat. No. 14485-1-AP; RRID:AB_2269787
p-Akt Thr308	Cell Signaling Technology	Cat. No. 9275; RRID:AB_329828
Akt	Cell Signaling Technology	Cat. No. 9272; RRID:AB_329827
p-eIF2α Ser51	Cell Signaling Technology	Cat. No. 9721; RRID:AB_330951
eIF2α	Cell Signaling Technology	Cat. No. 9722; RRID:AB_2230924
p-eEF2 Thr56	Cell Signaling Technology	Cat. No. 2331; RRID:AB_10015204
eEF2	Cell Signaling Technology	Cat. No. 2332; RRID:AB_10693546
MuRF1	ECM Biosciences	Cat. No. AP2041; RRID:AB_2208833
Atrogin-1	R&D Systems	Cat. No. AF5366; RRID:AB_2246979
LC3A/B	Cell Signaling Technology	Cat. No. 4108; RRID:AB_213770
GAPDH	Proteintech Group	Cat. No. 60004-1-Ig; RRID:AB_2107436
*Secondary*		
Goat anti-rabbit IgG (H + L)-HRP conjugate	Bio-Rad	Cat. No. 170-6515; RRID:AB_11125142
Goat anti-mouse IgG (H + L)-HRP conjugate	Bio-Rad	Cat. No. 170-6516; RRID:AB_11125547

eEF2, eukaryotic elongation factor 2; eIF2α, eukaryotic initiation factor 2α; eIF4E, eukaryotic initiation factor 4E; eIF4G, eukaryotic initiation factor 4 G; LC3A/B, microtubule associated protein light chain; mTOR, mechanistic target of rapamycin; MuRF1, muscle RING-finger protein 1; p, phospho; rag, ras-related GTP-binding protein; rheb, ras homolog enriched in brain; Sestrin2, stress response protein 2; S6K1, ribosomal protein S6 kinase 1; 4EBP1, eukaryotic initiation factor 4E-binding protein 1.

### mTOR·Ras Homolog Enriched in Brain, mTOR·Ras-Related GTP-Binding Protein (Rag) A, mTOR·RagC, and Stress Response Protein (Sestrin) 2·GTPase-Activating Protein Toward Rags2 Complex Abundance

Skeletal muscle was homogenized in CHAPS buffer (40 mmol/L HEPES, 120 mmol/L sodium chloride, 1 mmol/L EDTA, 10 mmol/L pyrophosphate, 10 mmol/L β-glycerophosphate, 40 mmol/L sodium fluoride, 1.5 mmol/L sodium vanadate, 0.3% wt/vol CHAPS, 1 mmol/L benzamidine, 1 mmol/L DTT, and 1% vol/vol protease inhibitor cocktail; pH 7.5). Following incubation on a platform rocker for 30 min at 4°C, lysates were centrifuged at 1,000 *g* for 10 min at 4°C and incubated with anti-Raptor (1:50; mTOR conjugates) or anti-Mios (1:50, Sestrin2·GATOR2 complex) overnight at 4°C. Immunoprecipitate was recovered with BioMag goat anti-rabbit IgG magnetic beads (Cat. No. 310207; Qiagen, Germantown, MD) blocked in 0.1% wt/vol nonfat dry milk in CHAPS buffer for 1 h at 4°C, washed twice with CHAPS buffer, and washed once with CHAPS buffer containing 60 mM HEPES and 200 mM sodium chloride. After recovery of the immunoprecipitate, the sample was resuspended in 100 µL 1× Laemmli buffer, boiled for 5 min, and separated by SDS-PAGE on triple-wide gels; 20 µL immunoprecipitated samples (for RagA, RagC, and Rheb analysis) and 10 µL (for mTOR and Mios analysis) were electrophoresed on separate gels. After wet transfer onto PVDF membranes, blots were blocked in 5% wt/vol nonfat dry milk in TBS-T for 1 h, incubated with anti-Rheb (1:1,000), anti-RagA (1:1,000), anti-RagC (1:1,000), anti-mTOR (1:1,000), anti-Sestrin2 (1:1,000), or anti-Mios (1:1,000) overnight at 4°C, incubated with the corresponding secondary antibody (1:5,000) for 1 h at room temperature, and visualized by chemiluminescence. Total Rheb, RagA, and RagC abundances were normalized to total mTOR abundance; total Sestrin2 abundance was normalized to total Mios abundance.

### Protein Immunoblot Analysis

Tissue (skeletal muscle or solid organs) was homogenized in seven volumes of homogenization buffer (20 mmol/L HEPES, 2 mmol/L EGTA, 50 mmol/L sodium fluoride, 100 mmol/L potassium chloride, 0.2 mmol/L EDTA, and 1% vol/vol protease inhibitor cocktail; pH 7.4). Lysates were subject to SDS-PAGE on triple-wide gels; 20–40 µg protein was loaded per lane. Following electrophoresis, proteins were transferred onto PVDF membranes, blocked, and incubated with primary and secondary antibodies, as described previously (19, 34). The following primary antibodies were used: total eIF4E-binding protein 1 (4EBP1), 1:1,000; phospho-4EBP1 Thr70, 1:1,000; total ribosomal protein S6 kinase 1 (S6K1), 1:1,000; phospho-S6K1 Thr389, 1:2,000; total Akt, 1:1,000; phospho-Akt Thr308, 1:1,000; total eukaryotic initiation factor 2α (eIF2α), 1:2,000; phospho-eIF2α Ser51, 1:2,000; total eukaryotic elongation factor 2 (eEF2), 1:2,000; phospho-eEF2 Thr56, 1:2,000; atrogin-1, 1:1,000; muscle RING-finger protein 1 (MuRF1), 1:1,000; microtubule-associated protein 1 A/1B light chain I/II (LC3-I/II), 1:2,000, and GAPDH, 1:5,000. The dilution of the corresponding secondary antibody was 1:10,000 for Akt, phospho-Akt, eIF2α, phospho-eIF2α, eEF2, phospho-eEF2, and GAPDH and 1:5,000 for other targets. Immunoreactivity was visualized with chemiluminescence. Total protein abundance was normalized for loading with GAPDH, and phosphoprotein abundance was normalized to the corresponding total protein abundance after stripping and reprobing the same membranes with nonphosphospecific antibodies.

### BrdU Labeling, Muscle Histology, and Muscle Morphometry

To estimate myonuclear accretion, 5-bromo-2′-deoxyuridine (BrdU; 25 mg·kg^−1^ iv; Calbiochem, Billerica, MA) was administered to all piglets every 12 h on *days 19* and *20* of the experiment, inclusive, for a total of four injections. After euthanasia, the LD muscle (between the third and fifth rib) was dissected, mounted on gum tragacanth, snap-frozen in liquid nitrogen-cooled isopentane, and stored at −80°C until further analysis. LD muscle was cryosectioned at 10 µm at −20°C perpendicular to the muscle fibers in a randomly selected subset of pigs (INT, *n* = 8, 3 male and 5 female; CONT, *n* = 6, 2 male and 4 female).

To identify subsarcolemmal BrdU+ myonuclei, LD muscle sections were fixed, permeabilized, and subjected to antigen retrieval, as described previously (35). Following incubation with Image-IT FX Signal Enhancer (Cat. No. I36933, Thermo Fisher Scientific), sections were blocked with 10% vol/vol normal goat serum (NGS) and 1% vol/vol bovine serum albumin in TBS-T for 30 min and incubated sequentially with antidystrophin (1:50) overnight at 4°C, goat anti-rabbit IgG, Alexa Fluor 488 (1:100) for 1 h at room temperature, anti-BrdU (2.5 µg/mL) for 30 min at room temperature, biotinylated goat anti-mouse IgG, cross-adsorbed (1:250) for 10 min at room temperature, and streptavidin-conjugated AF647 (1:200; Cat. No. S32357, Thermo Fisher Scientific) for 10 min at room temperature. See Table 5 for comprehensive antibody information for immunohistochemistry analysis. Nuclei were stained with 4′, 6-diamidino-2-phenylindole (DAPI, 300 nM) for 10 min at room temperature before applying SlowFade Gold Antifade (Cat. No. S36940, Thermo Fisher Scientific) and mounting cover slips.

**Table 5.** Antibody information for antibodies used in immunohistochemistry experiments

Antibody	Source	Identifier
Subsarcolemmal BrdU+ myonuclei		
Dystrophin (H-300)	Santa Cruz Biotechnology	Cat. No. sc-15376; RRID:AB_2091230
BrdU (clone G3G4)	DSHB	Cat. No. G3G4; RRID:AB_2618097
Goat anti-rabbit IgG (H + L) cross-adsorbed secondary antibody, Alexa Fluor 488	Thermo Fisher Scientific	Cat. No. A-11008; RRID:AB_143165
Goat anti-mouse IgG (H + L), biotinylated	Vector Laboratories	Cat. No. BA-9200; RRID:AB_2336171
Sublaminal Pax7+ nuclei		
Laminin	Millipore Sigma	Cat. No. L9393; RRID:AB_477163
Pax7	DSHB	Cat. No. PAX7; RRID:AB_2299243
Goat anti-rabbit IgG (H + L) highly cross-adsorbed secondary antibody, Alexa Fluor 488	Thermo Fisher Scientific	Cat. No. A-11034; RRID:AB_2576217
Biotin-SP goat anti-mouse IgG, Fcγ subclass 1 specific	Jackson ImmunoResearch Laboratories	RRID:AB_2338571

For sublaminal Pax7+ nuclei analysis, muscle sections were fixed, permeabilized, and subjected to antigen retrieval, as described previously (36). Following incubation with Image-IT FX Signal Enhancer, sections were blocked with 5% vol/vol NGS in PBS-T for 30 min and incubated sequentially with anti-Pax7 (8 µg/mL) and anti-laminin (1:100) overnight at 4°C, biotin-SP goat anti-mouse IgG, Fcγ subclass 1 specific (1:1,000) and biotinylated goat anti-mouse IgG, highly cross-adsorbed (1:1,000) for 45 min at room temperature, and streptavidin-conjugated AF647 (1:1,000) for 30 min at room temperature. Nuclei were stained with DAPI (300 nM) for 10 min at room temperature before applying SlowFade Gold AntiFade and mounting cover slips.

Section images were acquired with laser scanning confocal microscopy (Leica SP8 TCS, Leica Microsystems, Buffalo Grove, IL) and the LAS X software package (Leica Microsystems). DAPI was excited at 405 nm and detected between 430 and 480 nm; AF488 was excited at 488 nm and detected between 500 and 550 nm; AF647 was excited at 633 nm and detected between 650 and 750 nm. Maximum projection images were used for subsequent analysis. Approximately 1,600 fibers per muscle per pig were analyzed for each of the BrdU- and Pax7-stained sections (∼15 to 20 nonoverlapping sections). Myonuclei were defined as nuclei in which at least 50% of their circumference was located within a muscle fiber; SCs were defined as sublaminal Pax7+ nuclei. Muscle sections were also stained without anti-Pax7 or anti-BrdU primary antibodies to validate the specificity of the analysis (data not shown). Fiber cross-sectional area (CSA) and minimum Feret diameters were quantified with Image-Pro Plus software v. 6.2(MediaCybernetics, Silverspring, MD) from ∼800 fibers per pig. Myonuclear domain size was calculated as the quotient of mean fiber CSA and number of myonuclei per fiber.

### Statistical Analysis

Data were analyzed with the generalized linear mixed model procedure of SAS software (SAS 9.4, SAS Institute, Cary, NC). Body weight, body composition, protein synthesis, immunoblot, muscle morphometry, and muscle histology data were analyzed by one-factor ANOVA. Plasma insulin, glucose, and BCAA concentration data were analyzed by two-factor ANOVA; treatment and time were considered the main effects, and time was considered as a repeated measure. Simple main effects analysis was carried out to determine differences in insulin or substrates within time points. In these analyses, the individual pig was considered the experimental unit, and pig and litter were included as random effects to account for the correlation among piglets obtained from the same sow. Mean muscle fiber CSA, minimum Feret diameter, and the distribution of CSAs and minimum Feret diameters were analyzed by one-factor ANOVA. The effect of sex as a covariate was not significant in these analyses; thus, sex was omitted from the final statistical model and differences were not compared between males and females. The normality of residuals was assessed using the Shapiro-Wilk test statistic. Data are presented as least squares means ± standard error. Differences among treatments were determined with a Tukey’s post hoc test and were considered significantly different at *P* < 0.05.

## RESULTS

### Body Weight Gain and Body Composition

**Figure 1.**
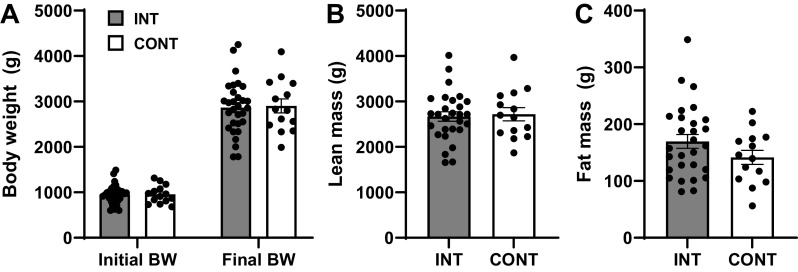
Initial and final body weights (*A*), lean mass (*B*), and fat mass (*C*) of preterm pigs provided intermittent bolus (INT; *n* = 30 for body weight, 7 male and 23 female; *n* = 28 for body composition, 7 male and 21 female) or continuous (CONT; *n* = 14, 5 male and 9 female) feeding for 21 days. Two pigs from the INT group were excluded from body composition analysis due to inadequate sedation for the dual-energy X-ray absorptiometry scan. Data were analyzed by one-factor ANOVA. Values are least squares means ± SE; individual data are shown.

Body weight increased over time in both INT and CONT groups. Initial and final body weight were not different between INT and CONT groups (Fig. 1*A*). There was no effect of feeding modality on lean mass (Fig. 1*B*), fat mass (Fig. 1*C*), or spine length (indicative of linear growth; data not shown).

### Plasma Glucose, Insulin, and Branched-Chain Amino Acid Concentrations

**Figure 2.**
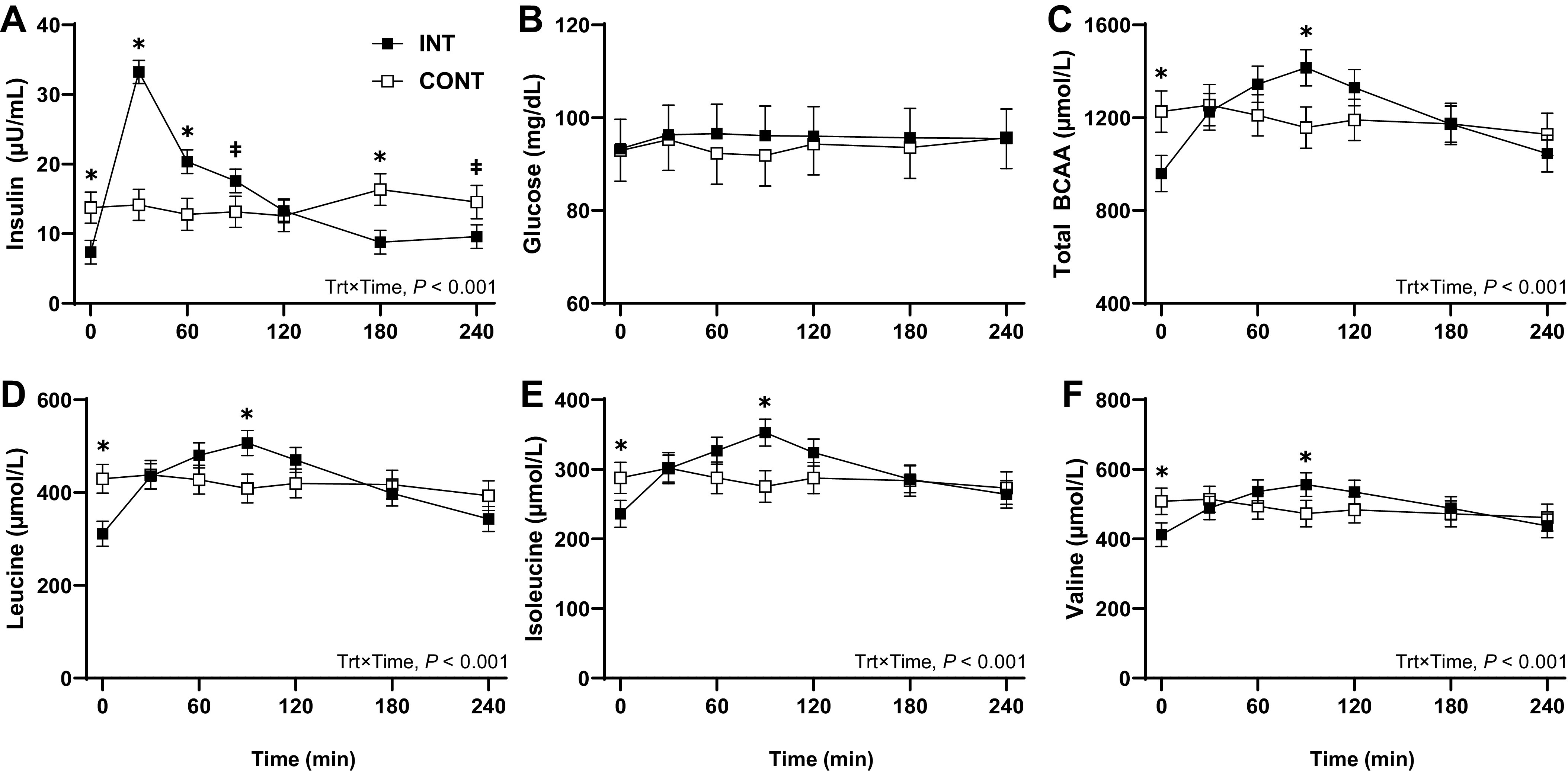
Plasma insulin (*A*), glucose (*B*), BCAA (*C*), leucine (*D*), isoleucine (*E*), and valine (*F*) concentrations over 4 h of preterm pigs provided intermittent bolus (INT; *n* = 27, 7 male and 20 female) or continuous (CONT; *n* = 12, 4 male and 8 female) feeding for 21 days. Three pigs from the INT group and two pigs from the CONT group were excluded from analysis due to nonpatent catheters for blood collection. Data were analyzed by two-factor ANOVA. Values are least squares means ± SE. *P < 0.05 and ‡*P* < 0.10, INT vs. CONT within time point. BCAA, branched-chain amino acid.

Plasma insulin concentration in the INT group peaked 30 min after feeding to ∼33.0 μU/mL and subsequently returned to prefeeding levels by 180 min after feeding (∼8.3 μU/mL, *P* < 0.001; Fig. 2*A*). The mean plasma insulin concentration for the CONT group was ∼13.5 μU/mL and remained steady over time. In the INT group, plasma insulin concentration was higher at 30 and 60 min (*P* < 0.01) and tended to be higher at 90 min (*P* = 0.08) compared with the CONT group. Plasma glucose concentration did not increase with feeding in the INT group (Fig. 2*B*). Plasma glucose was steady over time in the INT and CONT feeding modalities and was not different between groups. In the INT group, plasma BCAA concentrations increased 49% to ∼1,385 μmol/L with feeding and declined to prefeeding levels by 240 min after feeding (*P* < 0.001; Fig. 2*C*). Mean plasma BCAA concentrations were steady at ∼1,180 μmol/L in the CONT group. Plasma BCAA concentrations were 24% lower in the INT group before feeding at 0 min, and 21% higher after feeding at 90 min, compared with the CONT group (*P* < 0.05). The patterns for plasma Leu (Fig. 2*D*), Ile (Fig. 2*E*), and Val (Fig. 2*F*) concentrations over time and between INT and CONT feeding modalities were consistent with total BCAA concentrations.

### Tissue Fractional Protein Synthesis Rates

**Figure 3.**
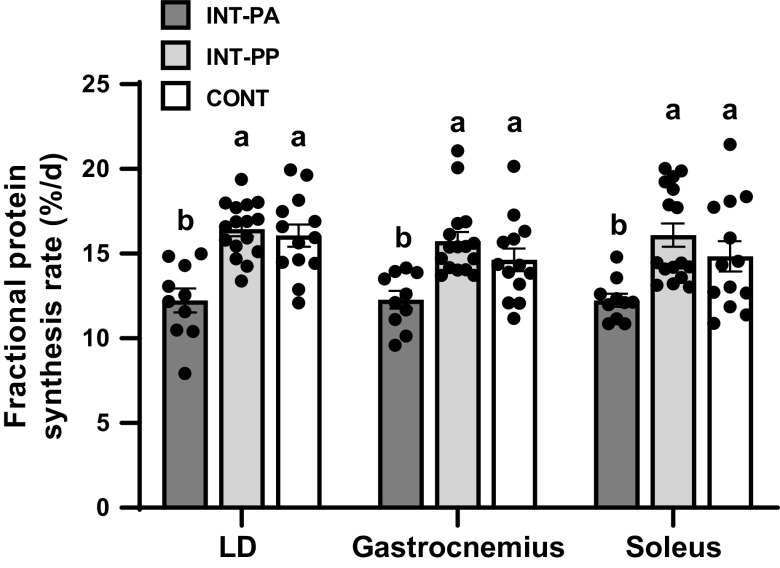
Fractional protein synthesis rate of the longissimus dorsi (LD), gastrocnemius, and soleus muscles of preterm pigs provided intermittent bolus (INT) or continuous (CONT) feeding for 21 days. Muscle from pigs fed by intermittent bolus feeding was sampled in the postabsorptive state (INT-PA, before feeding) and in the postprandial state (INT-PP, 60 min after feeding). Data were analyzed by one-factor ANOVA followed by the Tukey’s procedure. Three pigs from the INT-PA group and two pigs from the CONT group were excluded from analysis due to nonpatent catheters for tracer infusion and blood collection. Values are least squares means ± SE; individual data are shown; CONT, *n* = 12 (4 male and 8 female); INT-PA, *n* = 11 (2 male and 9 female); INT-PP, *n* = 16 (5 male and 11 female). Means without a common superscript letter differ, *P* < 0.05.

Compared with the INT-PA group, feeding increased protein synthesis by 34% in the LD, 28% in the gastrocnemius, and 31% in the soleus in the INT-PP group (*P* < 0.01; Fig. 3). In the LD and gastrocnemius muscles, protein synthesis was similar between the INT-PP and CONT groups, whereas in the soleus muscle, protein synthesis in the CONT group was intermediate between the INT-PA and INT-PP groups. Among organs, feeding increased protein synthesis in the heart by 21% and in the lung by 15% in the INT-PP group compared with the INT-PA group (*P* < 0.05; Table 6), whereas feeding did not increase protein synthesis in the brain, liver, jejunum, or pancreas. Protein synthesis was not different between the INT-PP and CONT groups among organs, except for the lung where protein synthesis was increased in the INT-PP compared with the INT-PA and CONT groups (*P* < 0.05).

**Table 6.** Tissue fractional protein synthesis rates (%/day) of preterm pigs fed by intermittent bolus or continuous feeding for 21 days

	Treatment			
Tissue	INT-PA	INT-PP	CONT	*P* value
Heart	13.7 ± 0.5^b^	16.7 ± 0.4^a^	15.7 ± 0.5^a^	<0.001
Lung	26.5 ± 1.0^b^	30.5 ± 0.8^a^	27.6 ± 0.9^b^	0.006
Brain	12.4 ± 0.8	13.3 ± 0.6	12.0 ± 0.7	0.41
Liver	58.4 ± 1.7	62.1 ± 1.4	60.4 ± 1.7	0.27
Jejunum	59.5 ± 3.5	67.8 ± 2.9	63.3 ± 3.3	0.19
Pancreas	82.4 ± 6.1	88.8 ± 4.8	91.8 ± 5.5	0.51

Values are least squares means ± SE calculated from one-factor ANOVA and differences among treatments were determined with a Tukey’s post hoc test; CONT, *n* = 12 (4 male and 8 female); INT-PA, *n* = 11 (2 male and 9 female); INT-PP, *n* = 16 (5 male and 11 female). Three pigs from the INT-PA group and two pigs from the CONT group were excluded from analysis due to nonpatent catheters for tracer infusion and blood collection. Labeled means in a row without a common superscript letter differ, *P* < 0.05. CONT, continuous feeding; INT-PA, intermittent bolus feeding in postabsorptive state (before feeding); INT-PP, intermittent bolus feeding in postprandial state (60 min after feeding).

### Insulin, Amino Acid, and mTORC1 Signaling Pathway Activation

**Figure 4.**
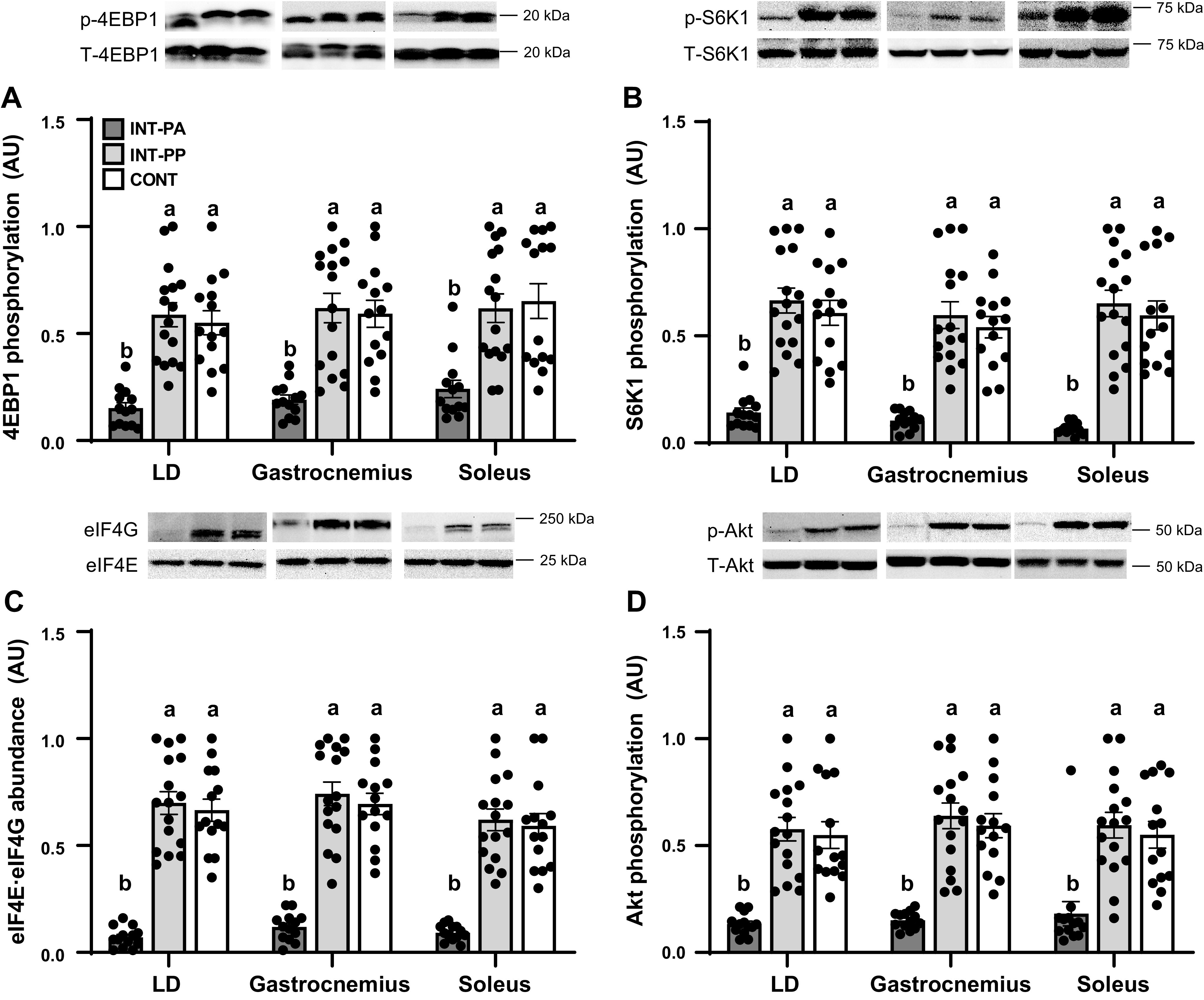
Relative abundance of phosphorylated Akt (*A*), phosphorylated eukaryotic initiation factor (eIF)4E-binding protein 1 (4EBP1; *B*), phosphorylated ribosomal protein S6 kinase 1 (S6K1; *C*), and the active eIF4E·eIF4G complex (*D*) in the longissimus dorsi (LD), gastrocnemius, and soleus muscles of preterm pigs provided intermittent bolus (INT) or continuous (CONT) feeding for 21 days. Phosphoprotein abundance was normalized to the corresponding total protein abundance; eIF4E·eIF4G abundance was normalized to total eIF4E abundance. Representative immunoblots are shown for each treatment group. Muscle from pigs fed by intermittent bolus feeding was sampled in the postabsorptive state (INT-PA, before feeding) and in the postprandial state (INT-PP, 60 min after feeding). Data were analyzed by one-factor ANOVA followed by the Tukey’s procedure. Values are least squares means ± SE; individual data are shown; CONT, *n* = 14 (5 male and 9 female); INT-PA, *n* = 13 (2 male and 11 female); INT-PP, *n* = 16 (5 male and 11 female). Means without a common superscript letter differ, *P* < 0.05. AU, arbitrary units.

**Figure 5.**
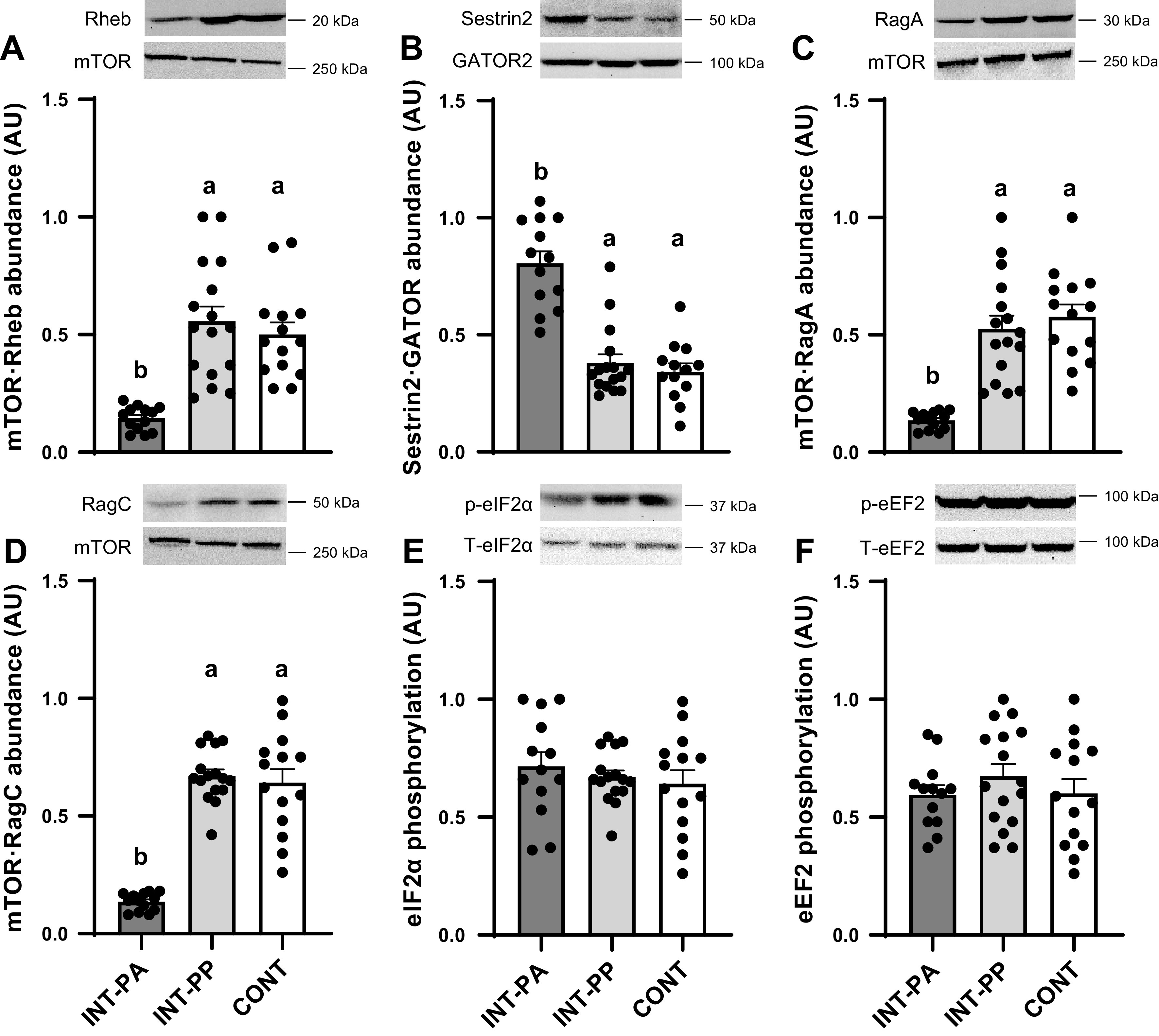
Relative abundance of the mechanistic target of rapamycin (mTOR) with Ras homolog enriched in brain complex (Rheb; *A*), Sestrin2·GATOR2 complex (*B*), mTOR·RagA complex (*C*), mTOR·RagC complex (*D*), phosphorylated eukaryotic initiation factor 2α (eIF2α; *E*), and phosphorylated eukaryotic elongation factor 2 (eEF2; *F*) in the longissimus dorsi (LD) muscle of preterm pigs provided intermittent bolus (INT) or continuous (CONT) feeding for 21 days. Phosphoprotein abundance was normalized to the corresponding total protein abundance; mTOR·Rheb, mTOR·RagA, and mTOR·RagC abundances were normalized to total mTOR abundance; Sestrin2·GATOR2 abundance was normalized to total Mios (GATOR2) abundance. Representative immunoblots are shown for each treatment group. LD muscle from pigs fed by intermittent bolus feeding was sampled in the postabsorptive state (INT-PA, before feeding) and in the postprandial state (INT-PP, 60 min after feeding). Data were analyzed by one-factor ANOVA followed by the Tukey’s procedure. Values are least squares means ± SE; individual data are shown; CONT, *n* = 14 (5 male and 9 female); INT-PA, *n* = 13 (2 male and 11 female); INT-PP, *n* = 16 (5 male and 11 female). One measurement for Sestrin2·GATOR2 (CONT group) and mTOR·RagA (INT-PP group) was excluded from analysis due to technical error. Means without a common superscript letter differ, *P* < 0.05. AU, arbitrary units.

To determine whether the similarities in tissue protein synthesis rates between INT and CONT pigs were consistent with mTORC1 signaling, we assessed the abundance and activation of components of the insulin, amino acid, and mTORC1 signaling pathways in the LD muscle. We also assessed a subset of components in other muscles and organ tissues. There were no differences in the total abundance of proteins involved in mTORC1 activation or translation initiation factor signaling in any tissue among treatment groups (data not shown). Downstream of mTORC1, feeding increased the phosphorylation of 4EBP1 (Thr70; Fig. 4*A*), S6K1 (Thr389; Fig. 4*B*), and the association of eIF4E with eIF4G (Fig. 4*C*) in the LD, gastrocnemius, and soleus muscles in the INT-PP group compared with the INT-PA group (*P* < 0.01). Feeding also increased the phosphorylation of Akt (Thr308; Fig. 4*D*) in the LD, gastrocnemius, and soleus muscles (*P* < 0.01). Upstream of mTORC1, feeding increased the association of mTOR with Rheb, which together with Akt phosphorylation are indices of insulin signaling, in the LD muscle in the INT-PP group compared with the INT-PA group (*P* < 0.01; Fig. 5*A*). Feeding also diminished the abundance of the inhibitory Sestrin2·GATOR2 complex (Fig. 5*B*) and promoted the association of mTOR with RagA and RagC (Fig. 5, *C* and *D*), indices of amino acid signaling, in the LD muscle in the INT-PP group compared with the INT-PA group (*P* < 0.01). However, the activation of the insulin, amino acid, and mTORC1 signaling pathways in all muscles examined was not different between the INT-PP and CONT groups. The phosphorylation of eIF2α (Ser51; Fig. 5*E*) and eEF2 (Thr56; Fig. 5*F*), which in part regulate translation initiation and elongation, respectively, was not different in the LD muscle among treatment groups.

**Figure 6.**
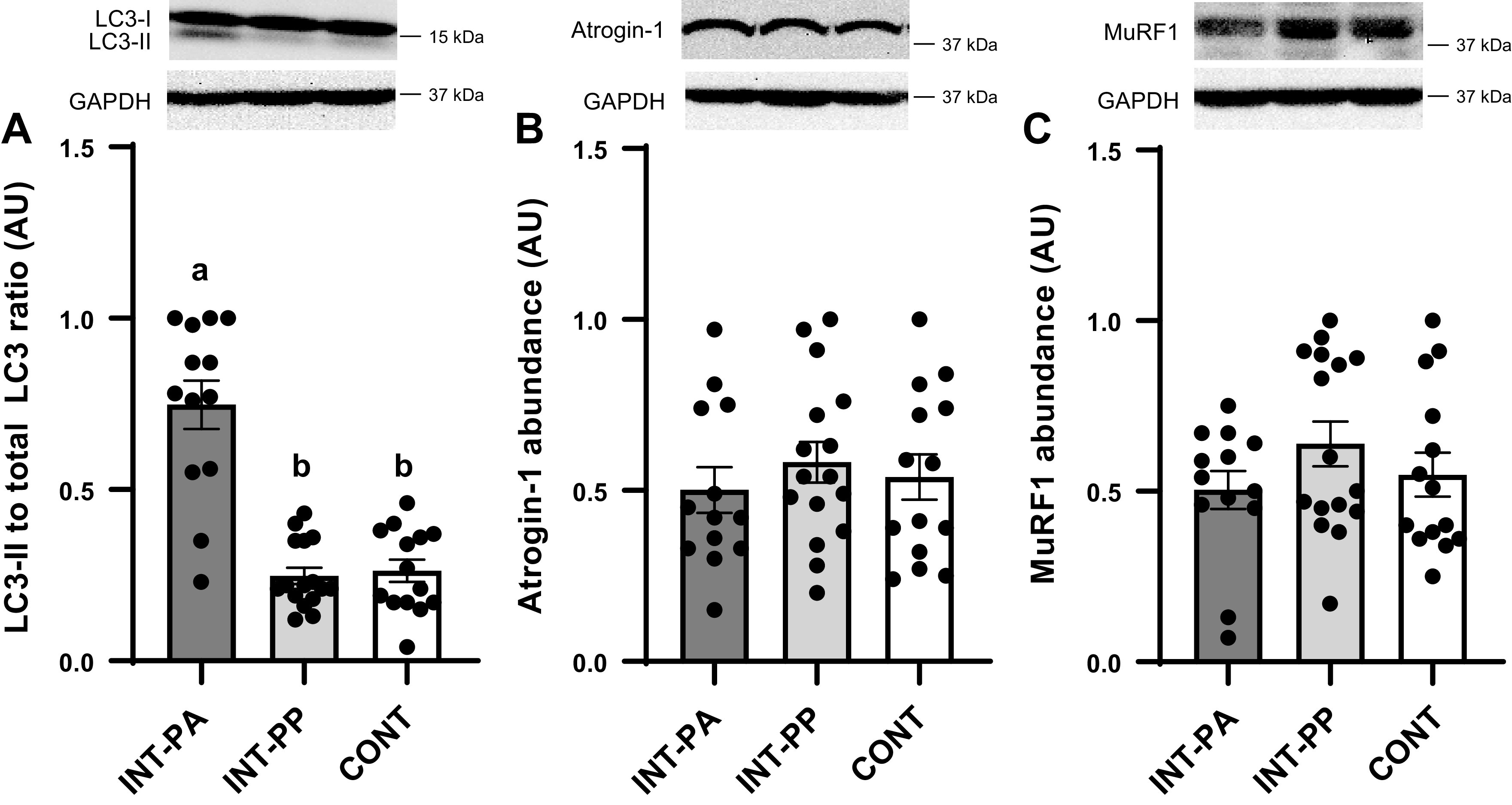
Ratio of microtubule-associated protein light chain 3 (LC3)-II to total LC3 (*A*), and relative abundance of atrogin-1 (*B*) and muscle RING-finger protein 1 (MuRF1; *C*) in the longissimus dorsi (LD) muscle of preterm pigs provided intermittent bolus (INT) or continuous (CONT) feeding for 21 days. Atrogin-1 and MuRF1 abundances were normalized to GAPDH abundance. Representative immunoblots are shown for each treatment group. LD muscle from pigs fed by intermittent bolus feeding was sampled in the postabsorptive state (INT-PA, before feeding) and in the postprandial state (INT-PP, 60 min after feeding). Data were analyzed by one-factor ANOVA followed by the Tukey’s procedure. Values are least squares means ± SE; individual data are shown; CONT, *n* = 14 (5 male and 9 female); INT-PA, *n* = 13 (2 male and 11 female); INT-PP, *n* = 16 (5 male and 11 female). Means without a common superscript letter differ, *P* < 0.05. AU, arbitrary units.

In the LD muscle, the LC3-II to total LC3 ratio, an index of autophagosome formation required for the degradation of cellular and organelle proteins, was reduced with feeding in the INT-PP group compared with the INT-PA group (*P* < 0.01) but was not different between the INT-PP and CONT groups (Fig. 6*A*). The abundance of the muscle-specific ubiquitin protein ligases atrogin-1 and MuRF1, needed for the degradation of myofibrillar proteins (37), in LD muscle was not different among treatment groups (Fig. 6, *B* and *C*).

Among organs, feeding increased the phosphorylation of Akt, 4EBP1, and S6K1 in the heart, and the phosphorylation of 4EBP1 in the lung, in the INT-PP group compared with the INT-PA group (*P* < 0.05; Table 7), whereas the phosphorylation of these proteins was not increased with feeding in the brain, liver, jejunum, or pancreas. The phosphorylation of Akt, 4EBP1, and S6K1 did not differ between the INT-PP and CONT groups.

**Table 7.** Relative abundance of phosphorylated signaling proteins in organs of preterm pigs fed by intermittent bolus or continuous feeding for 21 days

	Treatment			
Tissue	INT-PA	INT-PP	CONT	*P* value
Heart				
p-Akt	0.34 ± 0.06^b^	0.70 ± 0.05^a^	0.67 ± 0.06^a^	<0.001
p-4EBP1	0.26 ± 0.06^b^	0.57 ± 0.05^a^	0.52 ± 0.06^a^	<0.001
p-S6K1	0.29 ± 0.06^b^	0.56 ± 0.06^a^	0.55 ± 0.06^a^	0.005
Lung				
p-Akt	0.44 ± 0.07	0.60 ± 0.06	0.56 ± 0.07	0.19
p-4EBP1	0.43 ± 0.05^b^	0.60 ± 0.05^a^	0.57 ± 0.05^a^	0.049
p-S6K1	0.45 ± 0.08	0.58 ± 0.07	0.57 ± 0.07	0.36
Brain				
p-Akt	0.44 ± 0.06	0.47 ± 0.05	0.48 ± 0.06	0.87
p-4EBP1	0.59 ± 0.06	0.53 ± 0.05	0.57 ± 0.06	0.73
p-S6K1	0.47 ± 0.06	0.45 ± 0.06	0.49 ± 0.06	0.84
Liver				
p-Akt	0.63 ± 0.06	0.66 ± 0.05	0.63 ± 0.06	0.88
p-4EBP1	0.61 ± 0.06	0.69 ± 0.05	0.66 ± 0.05	0.63
p-S6K1	0.54 ± 0.07	0.55 ± 0.06	0.52 ± 0.07	0.93
Jejunum				
p-Akt	0.65 ± 0.07	0.60 ± 0.07	0.62 ± 0.07	0.89
p-4EBP1	0.54 ± 0.06	0.53 ± 0.06	0.49 ± 0.06	0.85
p-S6K1	0.41 ± 0.06	0.46 ± 0.05	0.44 ± 0.06	0.83
Pancreas				
p-Akt	0.50 ± 0.07	0.52 ± 0.06	0.56 ± 0.07	0.83
p-4EBP1	0.69 ± 0.05	0.72 ± 0.05	0.65 ± 0.05	0.63
p-S6K1	0.48 ± 0.06	0.46 ± 0.06	0.51 ± 0.06	0.82

Values are least squares means ± SE calculated from one-factor ANOVA and differences among treatments were determined with a Tukey’s post hoc test; CONT, *n* = 14 (5 male and 9 female); INT-PA, *n* = 13 (2 male and 11 female); INT-PP, *n* = 16 (5 male and 11 female). Labeled means in a row without a common superscript letter differ, *P* < 0.05. Phosphoprotein abundance was normalized to the corresponding total protein abundance. CONT, continuous feeding; INT-PA, intermittent bolus feeding in postabsorptive state (before feeding); INT-PP, intermittent bolus feeding in postprandial state (60 min after feeding); p, phospho; S6K1, ribosomal protein S6 kinase 1; 4EBP1, eukaryotic initiation factor 4E-binding protein 1.

### LD Muscle Histology and Morphometry

**Figure 7.**
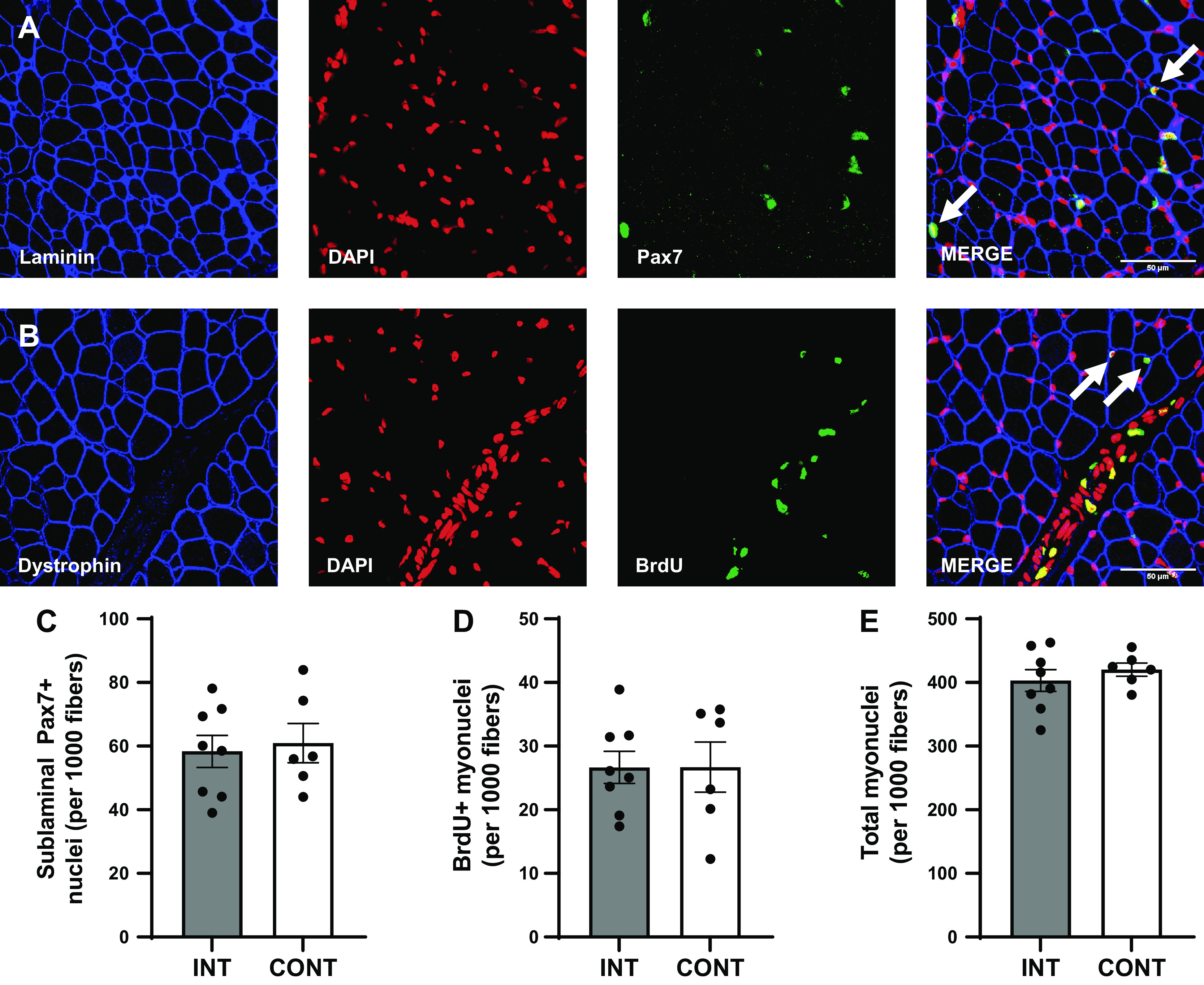
Representative longissimus dorsi (LD) muscle cross-sections stained for sublaminal Pax7+ nuclei (*A*); muscle cross-sections were stained for laminin (blue), nuclei (red; DAPI), and Pax7 (green). White arrows indicate sublaminal Pax7+ nuclei in the merged image; scale bar = 50 µm. Representative LD muscle cross-sections stained for subsarcolemmal 5-bromo-2′-deoxyuridine (BrdU)+ myonuclei (*B*); muscle cross-sections were stained for dystrophin (blue), nuclei (red; DAPI), and BrdU (green). White arrows indicate subsarcolemmal BrdU+ myonuclei in the merged image; scale bar = 50 µm. Sublaminal Pax7+ nuclei (satellite cells) per 1,000 fibers (*C*), subsarcolemmal BrdU+ myonuclei per 1,000 fibers (*D*), and total subsarcolemmal myonuclei per 1,000 fibers (*E*) in the LD muscle of preterm pigs provided intermittent bolus (INT; *n* = 8, 3 male and 5 female) or continuous (CONT; *n* = 6, 2 male and 4 female) feeding for 21 days. Nuclei were quantified from an average of 1,600 fiber cross-sections per pig. Data were analyzed by one-factor ANOVA. Values are least squares means ± SE; individual data are shown.

**Figure 8.**
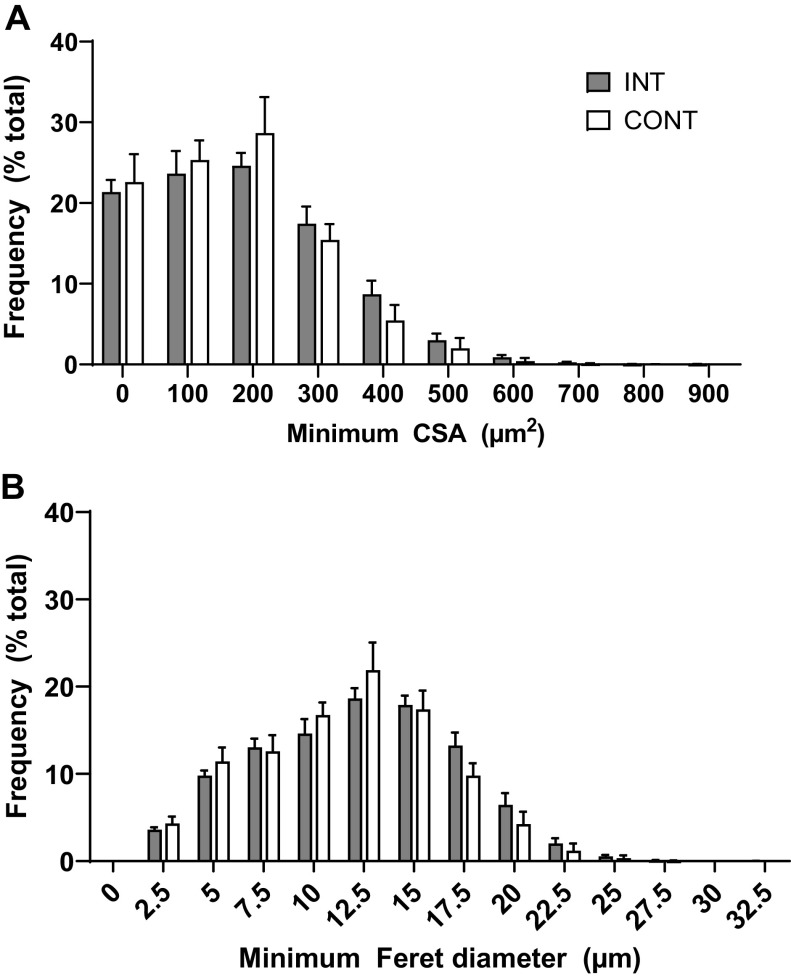
Distribution of longissimus dorsi (LD) muscle fiber cross-sectional areas (CSA) (*A*) and distribution of LD muscle fiber minimum Feret diameters (*B*) of preterm pigs provided intermittent bolus (INT; *n* = 8, 3 male and 5 female) or continuous (CONT; *n* = 6, 2 male and 4 female) feeding for 21 days. Each data point represents the frequency of fibers within each 100-µm^2^ bin (CSA) or each 2.5-µm bin (minimum Feret diameter). Fiber CSA and minimum Feret diameters were quantified from an average of 800 fiber cross-sections per pig. Data were analyzed by one-factor ANOVA. Values are least squares means ± SE.

In the LD muscle, there was no difference in the abundance of sublaminal Pax7+ SC (Fig. 7*A*), BrdU+ myonuclei (Fig. 7*B*), or total myonuclei (Fig. 7*C*) per 1,000 fibers between INT and CONT pigs. The proportion of BrdU+ myonuclei to total myonuclei (6.6 vs. 6.3 ± 0.8% for INT and CONT, respectively), BrdU+ myonuclei to sublaminal Pax7+ SCs (49.1 vs. 45.0 ± 8.0% for INT and CONT, respectively), and sublaminal Pax7+ SCs to total Pax7+ SCs (78.8 vs. 78.8 ± 2.2% for INT and CONT, respectively) was not different between groups. An estimate of muscle fiber size was derived from both mean CSA and minimum Feret diameter. Neither muscle fiber mean CSA (233 vs. 213 ± 12 µm^2^ for INT and CONT, respectively; Fig. 8*A*) nor muscle fiber minimum Feret diameter (13.5 vs. 12.8 ± 0.4 µm for INT and CONT, respectively; Fig. 8*B*) were different between groups. Myonuclear domain size also did not differ between groups (579 vs. 507 ± 31 µm^2^ per myonucleus for INT and CONT, respectively).

## DISCUSSION

Postnatal growth faltering is a common complication of premature birth (37). Skeletal muscle is particularly at risk for growth restriction, whereas adipose tissue growth is either maintained or increased (10, 38). Because such alterations in body composition may predispose infants born preterm to long-term morbidities including insulin resistance, type 2 diabetes, and cardiovascular disease, strategies to augment muscle protein accretion, the largest contributor to lean growth, while mitigating fat deposition need to be identified (39). This is especially relevant considering that insufficient nutrient intake in premature infants can impede adequate lean growth (6, 9). Altering feeding modality is one such possible approach. We determined previously that in neonatal pigs born at term, intermittent bolus feeding results in greater lean mass accretion than providing the same nutrient quantities as a continuous feed (19, 20, 22, 23). We attributed this to the intermittent feeding-induced surges in insulin and amino acids that, in turn, stimulate signaling pathways that regulate muscle protein synthesis. However, whether premature infants benefit from intermittent rather than continuous feeding remains an open question because clinical trials to date have yielded inconsistent results, in part due to small sample sizes, confounding factors, and protocol disruptions (40). In this study, we investigated how intermittent bolus and continuous feeding modalities impact muscle anabolic signaling and protein synthesis, SC abundance, myonuclear accretion, and body composition in a preterm neonatal pig model of the premature infant (27). A key motivation for this study was the observation that, although feeding, insulin, and amino acids all promote anabolic signaling and protein synthesis in skeletal muscle in pigs born preterm and at term, the magnitude of the response is blunted in preterm compared with term pigs (25, 26); if persistent, this could mitigate any advantage of intermittent feeding following premature birth.

In contrast to our hypothesis, in pigs born preterm, intermittent bolus feeding did not enhance body weight or lean mass gain more than continuous feeding. The absence of differences in mean LD muscle fiber CSA and minimum Feret diameter, both measures of muscle fiber hypertrophy that reflect cumulative differences in muscle protein accretion over time, corroborates the lack of an effect of feeding modality. Compared with the postabsorptive state, intermittent feeding incurred postprandial increases in plasma insulin and amino acid concentrations, 4EBP1 and S6K1 phosphorylation, eIF4E·eIF4G complex abundance, and protein synthesis in skeletal and cardiac muscle. However, intermittent feeding did not elicit a greater response in anabolic signaling or protein synthesis than continuous feeding. This contrasts with our previous report in pigs born at term (23). There are at least two possible explanations for the finding that anabolic signaling, protein synthesis, and muscle growth are not affected by intermittent feeding in pigs born preterm in the same way as pigs born at term. First, the magnitude of the postprandial increase in plasma insulin and amino acid concentrations with intermittent feeding was insufficient to increase mTORC1 signaling and protein synthesis to a greater extent than with continuous feeding. Second, skeletal muscle in pigs born preterm is resistant to the feeding-induced surge in insulin, amino acids, or both.

Although intermittent feeding increased skeletal muscle mTORC1 signaling compared with postabsorptive levels, mTORC1 signaling did not differ between continuous and intermittent feeding in the postprandial state. We detected no differences in insulin or amino acid signaling upstream of mTORC1 in LD muscle or in specific readouts of downstream mTORC1 signaling in LD, gastrocnemius, and soleus muscles. The lack of difference in muscle protein synthesis directly is consistent with the similar insulin and amino acid signaling protein activation between feeding modalities, even though plasma insulin concentrations peaked at ∼33 μU/mL with intermittent feeding compared with 13.5 μU/mL with continuous feeding. Plasma BCAA concentrations increased from ∼900 to 1,350 μmol/L with intermittent feeding, whereas BCAA remained steady at 1,150 μmol/L with continuous feeding. The time points at which plasma insulin and amino acid concentrations were maximized with intermittent feeding were discordant. Plasma insulin peaked 30 min after feeding, whereas plasma BCAA peaked 90 min after feeding. In pigs born at term, however, the disparity between time to peak insulin and BCAA concentrations was much less (23). It is possible that peak insulin and amino acid levels need to coincide for maximal stimulation of muscle mTORC1 signaling and protein synthesis and, thus, muscle protein accretion. Although the extent to which postprandial plasma BCAA concentrations was increased with intermittent compared with continuous feeding in pigs born preterm was similar to those measured previously in pigs born at term, it was considerably less for insulin. We have consistently reported a 10-fold difference in postprandial insulin concentrations between intermittent and continuous feeding in pigs born at term, whereas the difference was less than double in pigs born preterm in the current study. We did not anticipate this difference in insulin release considering that the diet in the current study contained more lactose, protein, and fat than in previous long-term feeding studies in pigs born at term (23). This suggests that premature birth in pigs may impair pancreatic insulin secretion or that pancreatic insulin secretion is less responsive to substrate stimulation. This possibility is also supported by observations in sheep in which preterm birth has been associated with smaller pancreatic β cell mass, insulin mRNA expression, and lower insulin secretion in response to glucose stimulation at the equivalent age of 4 wk after term birth (41). Conversely, plasma insulin concentrations during continuous feeding in the current study were higher than in term pigs in previous studies (19, 20, 22, 23). Although insulin above 10 μU/mL is sufficient to induce maximal rates of skeletal muscle protein synthesis in neonatal pigs born at term, it is unclear whether this concentration is also sufficient to elicit a similar response in pigs born preterm (12, 13). Altered insulin secretion may affect the absolute insulin levels observed in response to intermittent and continuous feeding and thus contribute to the absence of a difference in insulin-induced mTORC1 activation and protein synthesis in skeletal muscle between the feeding modalities.

Alternatively, prematurity may blunt the capacity of skeletal muscle and other tissues to increase protein synthesis in response to insulin, amino acids, or both. We previously reported that feeding promotes protein synthesis in all tissues of both preterm and term pigs, but that prematurity blunts the response in skeletal and cardiac muscles (25). Compared with full-term pigs, prematurity produced a marked reduction in LD muscle insulin and amino acid signaling to mTORC1 after feeding. More recently, we showed that mTORC1 signaling and protein synthesis in skeletal muscle are blunted in response to equivalent and sustained increases in both insulin and amino acids in preterm compared with term pigs during hyperinsulinemic and hyperaminoacidemic clamps (26). This blunted insulin- and amino acid-induced mTORC1 activation was associated with reduced Akt phosphorylation and mTOR·Rag association, respectively, in muscle of preterm compared with term pigs. Preterm baboons born at 67% gestation likewise had impaired peripheral insulin sensitivity and reduced muscle and hepatic Akt phosphorylation relative to baboons born at term when both groups were studied in the first week of postnatal life (42, 43). The need for a higher rate of insulin infusion to normalize blood glucose in extremely low birth weight premature infants compared with low birth weight infants also is suggestive of insulin resistance (44). However, these responses were measured within several days of birth and do not necessarily reflect potential long-term impacts of prematurity on the capacity of skeletal muscle or other tissues to respond to insulin and amino acids. The lack of enhanced muscle insulin and amino acid signaling and protein synthesis in intermittently compared with continuously fed pigs in the current study is consistent with the possibility that skeletal and cardiac muscles in pigs born preterm remain relatively resistant to the surge in insulin and amino acids elicited with intermittent feeding during early postnatal life (i.e., at 12 days term-corrected age). In pigs born at term, intermittent feeding promotes Akt phosphorylation in LD muscle compared with continuous feeding (23), whereas it failed to do the same in pigs in the current study. Moreover, the abundance of the inhibitory Sestrin2·GATOR2 complex, a putative sensor for intracellular Leu concentrations (45), and the association of mTOR with Rag proteins were similar between intermittent and continuous feeding in this study. This suggests that muscle in the preterm neonate has a persistent deficit in the capacity to respond to a surge in insulin and amino acids produced by intermittent feeding, impeding its ability to accrue body protein at the same rate as a term neonate.

In the current study, eIF2α phosphorylation, which negatively regulates translation initiation, was not different among treatments in LD muscle. Conversely, eIF2α phosphorylation was decreased after 3 wk, but not 24 h, of intermittent feeding in term pigs, suggesting that eIF2α is not adversely affected by preterm birth (22, 23). There was also no difference in eEF2 phosphorylation in LD muscle among treatments in either preterm pigs in the current study or term pigs in previous studies. Together, the data indicate that impairment of protein synthesis induced by prematurity is independent of eIF2α and eEF2.

The regulation of protein degradation, along with the regulation of protein synthesis, determines muscle protein accretion. Muscle protein degradation occurs through three major proteolytic systems: the calpain system, which mediates the dissociation of sarcomeric proteins from intact myofibrils; the ubiquitin-proteasome system, which tags and degrades sarcomeric proteins; and the autophagy-lysosome system, which degrades the bulk cytosolic and membrane proteins (37). In the current study, the abundance of atrogin-1 and MuRF1, muscle-specific ubiquitin protein ligases that conjugate ubiquitin to sarcomeric proteins before degradation by the proteasome, did not differ in LD muscle among treatments in pigs born preterm. Considering that atrogin-1 and MuRF1 expression are crucial determinants of muscle protein degradation in catabolic states, we anticipated that their abundance would be unaffected by feeding modality in the growing neonate (46, 47). The autophagy-lysosome system, however, is more sensitive to feeding and nutrient status than the ubiquitin-proteasome system (19, 23, 48). The LC3-II to total LC3 ratio, an index of autophagosome formation and positive regulator of autophagy, was reduced with intermittent feeding in the postprandial compared with the postabsorptive state, but it was not different between intermittent and continuous feeding in LD muscle. Collectively, alterations in the regulation and rate of protein degradation in muscle are unlikely to affect lean growth in pigs fed intermittently or continuously.

Skeletal muscle growth is dependent on the coordinated activation of protein synthesis and the accretion of myonuclei derived from the proliferation, differentiation, and fusion of satellite cells into muscle fibers (16). Despite the clear role for specific nutrients and insulin in promoting mTORC1 activation and protein synthesis in skeletal muscle (49, 50), less is known about their role in regulating myonuclear accretion. Previously, we showed that restricting the protein intake of neonatal pigs reduces myonuclear accretion (35), and that Leu supplementation does not restore SC abundance or myonuclear accretion when protein intake is limiting (51). Although this implies an essential role for dietary protein in supporting myonuclear accretion in addition to protein synthesis in muscle, it is unclear whether time-dependent differences in insulin and substrate availability between intermittent and continuous feeding patterns over an extended period can affect this process. In this study, the abundance of total myonuclei, BrdU+ myonuclei, and Pax7+ SCs did not differ between feeding modalities, suggesting that the pattern of hormone and substrate supply associated with feeding did not affect myonuclear accretion in pigs born preterm that received adequate nutrient intake. The similar proportion of BrdU+ myonuclei to sublaminal Pax7+ SCs also implies that SC differentiation and fusion were not affected by feeding modality.

Protein accretion in organs, albeit to a lesser extent than in skeletal muscle, contributes to postnatal lean growth (52). Although both plasma insulin and amino acid concentrations increase protein synthesis in skeletal muscle, amino acids are more potent regulators of protein synthesis in other organs (32). Apart from the lung, we did not observe any difference in protein synthesis in the heart, brain, liver, jejunum, or pancreas between prolonged intermittent bolus and continuous feeding, nor did we observe an increase in protein synthesis in the brain, liver, jejunum, or pancreas before and after feeding in the intermittent group. The association between protein synthesis and markers of mTORC1 activation, 4EBP1 and S6K1 phosphorylation, was largely consistent in these tissues. In the lung, however, phosphorylation of 4EBP1 and S6K1 did not differ between feeding modalities despite increased lung protein synthesis with intermittent bolus feeding. The specific signaling basis for the observed increase in lung protein synthesis with intermittent bolus feeding is not readily apparent. In pigs born at term, we recently reported that prolonged intermittent bolus feeding increased liver protein synthesis compared with continuous feeding (24). In pigs born preterm, however, liver protein synthesis was not affected by feeding modality. Although the small separation in peak plasma amino acid concentrations between intermittent bolus and continuous feeding modalities in pigs born preterm may underpin the lack of a difference in liver protein synthesis, it is not clear why the rise in amino acids after feeding in the intermittent group did not stimulate liver protein synthesis.

In summary, prolonged intermittent bolus feeding does not enhance body weight gain or lean mass more than continuous feeding in pigs born preterm. This contrasts with our previous study in pigs born at term that reports more rapid lean growth with intermittent compared with continuous feeding (23). Although plasma insulin and amino acid concentrations in pigs fed continuously may have been sufficient to promote mTORC1 activation and protein synthesis in skeletal muscle to the same extent as intermittent bolus feeding, it is also possible that the impaired feeding-induced increase in mTORC1 signaling and protein synthesis that we have documented in pigs shortly following premature birth persists during early postnatal life (25). For example, at equivalent term-corrected age, pigs born preterm in the current study weighed over 10% less and had 60% lower muscle protein synthesis rates than pigs born at term despite similar nutrient intake on a body weight basis (53). Although preterm and term pigs were not compared directly in this study, this supports the possibility that a relative anabolic resistance to feeding persists after preterm birth and may account for the deficits in lean mass that many premature infants exhibit into adulthood which impacts their lifelong metabolic health (3). Future studies are warranted to discern whether the preterm neonate is chronically resistant to the anabolic effects of insulin, amino acids, or both and to identify the mechanism responsible for this deficiency.

## GRANTS

This work is a publication of the USDA/ARS Children’s Nutrition Research Center, Department of Pediatrics, Baylor College of Medicine. Financial support for this project was provided by National Institute of Child Health and Human Development Grants HD-085573, HD-072891, HD-099080, USDA Current Research Information System Grant 3092–51000-060, and USDA National Institute of Food and Agriculture Grant 2013–67015-20438. The contents of this publication do not necessarily reflect the views or policies of the USDA, nor does the mention of trade names, commercial products, or organizations imply endorsement by the US Government.

## DISCLOSURES

No conflicts of interest, financial or otherwise, are declared by the authors.

## AUTHOR CONTRIBUTIONS

D.G.B., M.L.F., and T.A.D. conceived and designed research; M.R., J.K.N., A.S., H.V.N., B.S., C.C.S., M.A.V., O.O.O., and T.A.D. performed experiments; M.R., A.S., H.V.N., and T.A.D. analyzed data; M.R., J.K.N., A.S., M.L.F., and T.A.D. interpreted results of experiments; M.R. prepared figures; M.R. and T.A.D. drafted manuscript; M.R., J.K.N., A.S., D.G.B., M.L.F., and T.A.D. edited and revised manuscript; M.R., J.K.N., A.S., H.V.N., B.S., C.C.S., M.A.V., O.O.O., D.G.B., M.L.F., and T.A.D. approved final version of manuscript.
